# Prevalence and associated factors of postpartum depression among postpartum mothers in central region, Eritrea: a health facility based survey

**DOI:** 10.1186/s12889-020-09676-4

**Published:** 2020-10-27

**Authors:** Nahom Kiros Gebregziabher, Tesfit Brhane Netsereab, Yerusalem Gebremeskel Fessaha, Feven Andebrhan Alaza, Nardos Kidane Ghebrehiwet, Aman Hadish Sium

**Affiliations:** School of Public Health, Asmara College of Health Sciences, Asmara, Eritrea

**Keywords:** Postpartum depression, Asmara, Eritrea, DSM-V, Postpartum mothers

## Abstract

**Background:**

Postpartum depression (PPD) is a mood disorder that occurs within the first 12 months after delivery. It affects 20 to 40% of women living in the low-income countries. In resource limited countries discovering the predictors of PPD is important as it allows close follow-up and targeted screening of at risk mothers. The objective of this study was to assess the magnitude and predictors of PPD among recently delivered mothers in Central Region of Eritrea.

**Methods:**

This study used analytical cross-sectional study design to evaluate the magnitude of and factors associated with postpartum depression among 380 randomly selected mothers. The study was conducted in four primary health care facilities of Zoba Maekel (Central Region), Eritrea. A structured closed-ended questionnaire was used to capture the socio-demographic and maternity related information of the study participants. The standard Diagnostic and Statistical Manual of Mental Disorders Fifth Edition was used to assess depression. The dependent variable for this study was status of the mother with regard to PPD. The socio-demographic and maternity related variables of the mothers, presumed to influence the likelihood of developing postpartum depression, were the independent variables.

**Results:**

In this study the prevalence of PPD was found to be 7.4%. Mother’s who are housewives were less likely to develop PPD (AOR = 0.24, 95% CI: 0.06–0.97; *p* = 0.046), whereas, mothers with perceived low economic status (AOR = 13.33, 95% CI: 2.66–66.78; *p* = 0.002), lack of partner support (AOR = 5.8, 95% CI: 1.33–25.29; *p* = 0.019), unplanned pregnancy (AOR = 3.39, 95% CI: 1.24–9.28; *p* = 0.017), maternal illness after delivery (AOR = 7.42, 95% CI: 1.44–34.2; *p* = 0.016), and reside in Southwest-Asmara (AOR = 6.35, 95% CI: 1.73–23.23; *p* = 0.05) had statistically significant higher odds of postpartum depression.

**Conclusions:**

In the current study setting, factors that associated with PPD are grouped in to two domains; the woman’s potential to bear the forthcoming responsibility and the social support they get after delivery. The findings of this study imply the need to introduce an active screening program for PPD the health facilities as part of the postpartum care.

## Background

Postpartum depression (PPD) is a mood disorder that manifests with several somatic and emotional symptoms [1] within the first 12 months after delivery [2]. It is estimated that 20 to 40% of women living in the low-income countries experience depression during pregnancy or the postpartum period [3]. The prevalence of PPD shows a wide variation, affecting 8–50% of postnatal mothers, across countries [4, 5]. The reason for the variation in reported prevalence of PPD has been attributed to the differences in health-seeking behavior, and the trans-cultural variations in interpreting the symptoms [6, 7].

Existing evidence shows that PPD has negative effects on the mother-infant relationship [8], and child growth and development [9]. Hence, prompt identification and management of PPD is crucial in improving the general maternal health [10] and reducing infant and child mortality [11]. However, despite its common occurrence, postpartum depression often goes undiagnosed in many settings [9].

Eritrea is a country situated in the Horn of Africa. The country is divided into six regions namely Anseba, Debub, Debubawi Keih Bahri, Gash-Barka, Maekel, and Semenawi Keih Bahri. The population of the country is divided into nine ethnic groups which are culturally diverse. About 65% of the people reside in the rural areas and depend on subsistence farming for a living. The number of health facilities providing maternal and child health (MCH) services has been increasing, which is accompanied by increases in facility delivery. However, there is a marked difference on the rate of MCH service utilization across regions [12]

In Eritrea, a national KAP survey conducted by the Ministry of Health, in 2014, reported the prevalence of common mental disorders to be 14.5% [13]. In Eritrea the expected prevalence of common mental disorders is high [14], however, the actual incidence and prevalence reports are very low. This discrepancy is attributed to the underreporting of patients and under/misdiagnosis due to the lack of adequate number of health professionals trained in the field [15]. This suggestion is supported by findings from the KAP study, which reported that only 20% of the household respondents were able to identify signs and symptoms of mental disorders, and lack of adequate knowledge on the part of health workers [13]. The Mental Health Policy and Strategic Plan of the Ministry recognize a 97.7% treatment gap for mental problems in the country [16].

The exact cause of PPD is not well understood. However, factors like hormonal changes during pregnancy and after childbirth, genetic predisposition, birth related trauma, and other psycho-social and demographic factors have been hypothesized as potential risk factors [9, 17–19]. Several studies have tried to identify setting-specific determinants of postpartum depression among mothers. For instance, Kerie et al. identified age of the mother, unplanned pregnancy, chronic illness, death of infant, and current marital problem as predictors of postpartum depression in mothers from Southwest Ethiopia [20]. A study from Kars city, Turkey reported place of residence, mother’s occupational status, household income and history of psychiatric disease to be significant determinants of PPD [21]. Social support, stressful life events, mode of delivery, education, employment, and chronic health problems were significantly predictors of PPD in Lebanon [22]. On the other hand, a study from Turkey found mode of delivery to have no significant impact on the development of postnatal depression [23]. Studies have also tried to link PPD with breastfeeding [24–26], although methodological flaws made it difficult to draw conclusions [27]. All these findings call for systematic assessment of presumed potential predictors of PPD.

The global strategy for women’s, children’s and adolescents’ health (2016–2030) recommends screening and management of postpartum depression as one of the important postnatal interventions [28]. In resource-limited countries such as Eritrea, where there are no adequate mental health professionals, discovering the predictors of PPD is important as it allows close follow-up and targeted screening of at-risk mothers. Being able to identify mothers at risk of developing postpartum depression will lead to timely referral for diagnosis and proper treatment of the condition [19, 29]. This, in turn, will contribute to improvements in the maternal and child health outcomes [10]. Moreover, similar to other mental disorders, the magnitude of PPD is not known in the current study setting. The objective of this study was to assess the prevalence and predictors of PPD among recently delivered mothers in Central Region of Eritrea.

## Methods

### Study design

This study used analytical cross-sectional study design to evaluate the magnitude of and factors associated with postpartum depression.

### Study setting and population

This research was part of a study which was conducted to validate the Edinburgh Postnatal Depression Scale in Eritrea. The study was conducted in four primary health care facilities of Zoba Maekel (Central Region), Eritrea. All of the health facilities in the Region were categorized in to two groups, based on rural and urban setting, from which two health facilities were selected from each setting using simple random sampling technique. Accordingly, Tsaeda-kristian and Seregeka Health Centers were selected from the rural health facilities, and Semenawi Asmara Health Center and Freselam Health Station were selected from health facilities within the city of Asmara. These health facilities are meant to provide primary health care services like immunization, family planning, antenatal care and other basic health services, which make them ideal places to encounter postpartum women in our setting. Hence, the study was purposively conducted in primary health care facilities. The target population for this study was recently delivered mothers within 2 to 14 weeks postpartum. Mothers confirmed to be free from any known chronic illness and mental illnesses were included in the study.

### Sample size and sampling technique

The sample size for this study was determined by using the single population proportion formula [n = (z^2^ (P *(1-p))/d^2^]. Where: z-score value = 1.96, p (the proportion of postpartum depression) was taken as 50% (0.5), and margin of error (d) was set to be at 5% (0.05). Based on these parameters the initial sample size (n_1_) was 384. We used a correction factor [n_2_ = (n_1_*N)/ (N + n_1_))] to adjust the sample size to the actual population size (N), which was estimated to be 17,369. Based on this n_2_ was found to be 375. Finally, 5% of the sample was added for the expected non-response. Therefore, the final sample size was 393. The sample size was allocated to the selected health facilities based on probability proportional to the size, size being the number of mothers attending the health facilities for child immunization program. Then the researchers developed a sampling frame of eligible mothers, those who were present during the data collection period, in each health facility and study participants were selected by using simple random sampling technique.

### Data collection tools and techniques

A structured closed-ended questionnaire was developed to be used as data collection tool to capture the socio-demographic and maternity-related information of the study participants. The standard Diagnostic and Statistical Manual of Mental Disorders Fifth Edition (DSM-5) [2] was used to assess depression in the postpartum women. DSM-5 is an international diagnostic manual for mental disorders developed by the American Psychiatric Association and WHO in collaboration with other institutions and professionals working in the field of mental health. This manual has ten-item diagnostic criteria for Major Depressive Disorders (MDD). Accordingly the presence of five or more of the following symptoms during 2-weeks period, which represent a change from previous functioning, qualifies for the diagnosis of major depressive symptoms. This manual is definitive criteria for the diagnosis of MDD in the study setting and it requires a qualified professional to administer it.

First, the participants were interviewed, in a private room within the health facility, for the socio-demographic and other maternity related questions. Then DSM-5 interview for MDD was conducted by experienced qualified psychiatric-nurse in a separate room. Based on the diagnostic interview the mothers were categorized as depressed and not-depressed. Mothers diagnosed as having depression during the MDD interview were offered counseling on how to proceed with their condition.

### Outcome and predictor variables

The dependent variable for this study was status of the mother with regard to postpartum depression, as diagnosed by the DSM-5’s criteria. The socio-demographic and maternity related variables presumed to influence the likelihood of developing PPD, were the independent variables. The main maternity-related data included; gravidity, gestational age at delivery (term/preterm), mode of delivery, place of delivery, and postpartum days of the mother.

### Data analysis

Data was cleaned and entered into the Statistical Package for Social Sciences (SPSS) version 20 for analysis. Descriptive statistics (frequencies, proportions, means and standard deviations) were used to describe the socio-demographic and other maternity-related variables. In addition, chi-square test was used to assess the statistical significance of the observed difference in proportions. Bivariate and multivariate logistic regression analysis was conducted to identify the magnitude of the relationship between the dependent and independent variables. *P* value less than or equal to 0.05 was taken as indicator of statistical significance.

## Results

A total of 393 postpartum mothers were approached of which 380 were assessed for postpartum depression. Hence, the response rate was 96.6%. The prevalence of PPD among the study participants was found to be 7.4%. The mean age of the participants was 27.7 years (SD ± 5.23), with minimum and maximum age of 16 and 43 years, respectively. Most of the mothers were married (90.8%) and Christians (77.1%). A majority (76.1%) had secondary-level education and 77.9% were employed. Only 6.8% perceived their economic status as “low.” The number of children reported by most of the participants was between 1 and 3, and 92.9% reported having good support from partner or father of the baby. Presence or history of mental illness in close relatives was reported by 7.1%. Most (61.3%) of the study participants were from urban settings, with 39.5% from Northeast Asmara and 21.8% from Southwest Asmara. Chi-square analysis revealed statistically significant differences in proportions between participant’s depression status and marital status, occupation, economic status, availability of partner support, as well as residence (Table 1).

**Table 1:** Socio-demographic characteristics of the participants (*N* = 380)

Variable	Postpartum depression		Total N (%)	*p*-value
	No: n (%)	Yes: n (%)		
**Total**	352 (92.6)	28 (7.4)		
**Age category in years**				
16–24	94 (91.3)	9 (8.7)	103 (27.1)	0.638
25–34	214 (92.6)	17 (7.4)	231 (60.8)	
35–43	44 (95.7)	2 (4.7)	46 (12.1)	
**Marital Status**				
Single	29 (82.9)	6 (17.1)	35 (9.2)	0.02
Married	323 (93.6)	22 (6.4)	345 (90.8)	
**Religion**				
Christian	270 (92.2)	23 (7.8)	293 (77.1)	0.51
Muslim	82 (94.3)	5 (5.7)	87 (22.9)	
**Educational level**				
No education	5 (83.3)	1 (16.7)	6 (1.6)	0.218
Primary level	36 (85.7)	6 (14.3)	42 (11.1)	
Secondary level	270 (93.4)	19 (6.6)	289 (76.1)	
Tertiary level (12^+^)	41 (95.3)	2 (4.7)	43 (11.3)	
**Employment status**				
Unemployed	22 (73.3)	8 (26.7)	30 (7.9)	0.002
Employed	50 (92.6)	4 (7.4)	54 (77.9)	
Housewife	280 (94.6)	16 (5.4)	296 (14.2)	
**Perceived economic status**				
Low	16 (61.5)	10 (38.5)	26 (6.8)	< 0.001
Average	240 (94.9)	13 (5.1)	253 (66.6)	
Good	96 (95.0)	5 (5.0)	101 (26.6)	
**Partner support**				
No	21 (77.8)	6 (22.2)	27 (7.1)	0.002
Yes	331 (93.8)	22 (6.2)	253 (92.9)	
**Number of children**				
1–3	276 (92.0)	24 (8.0)	300 (78.9)	0.56
4–6	68 (94.4)	4 (5.6)	72 (18.9)	
7–11	8 (100)	0 (0)	8 (2.1)	
**Residence**				
Rural	138 (93.3)	9 (6.1)	147 (38.7)	0.46
Urban	214 (91.8)	19 (8.2)	233 (61.3)	
**History of mental illness in close relatives**				
Do not know	19 (79.2)	5 (20.8)	24 (7.1)	0.086
No	308 (93.6)	21 (6.4)	329 (86.6)	
Yes	25 (92.6)	2 (7.4)	27 (6.3)	
**Residence**				
Northeast Asmara	143 (95.3)	7 (4.7)	150 (39.5)	0.045
Berik Sub-zone	107 (93.9)	7 (6.1)	114 (30.0)	
Southwest Asmara	71 (85.5)	12 (14.5)	83 (21.8)	
Serejeka Sub-zone	31 (93.9)	2 (7.4)	33 (8.7)	

Almost one- third (30%) of the mothers were primigravida during the last pregnancy and 1.3% had been pregnant more than eight times (Table 2). Among those mothers who have been pregnant multiple times, 21.4% had a history of miscarriage. For 19.4% of the mothers the last pregnancy was unplanned, and 8.2% reported having had a preterm delivery, during past pregnancies. Only 8.2% of the mothers reported to have experienced complications either during pregnancy of childbirth. In this study proportion of home delivery was 5%, with majority (83.9%) of the mothers giving birth through normal vaginal delivery. Infant illness (any illness) and maternal illness, after delivery, was reported by 12.1 and 6.8% of the mothers, respectively. At the time of data collection the majority of the infants (96.3%) were exclusively breastfed. On chi-square analysis it was found that mothers who had unplanned pregnancy, those who experienced complications during pregnancy or childbirth, those who became ill after delivery, and those whose infants became sick to have statistically significant higher proportion of postpartum depression.

**Table 2:** Pregnancy and delivery-related characteristics of the participants (*N* = 380)

Variable	Postpartum depression		Total (%)	*p*-value
	No	Yes		
Total	N (%)	N (%)	N (%)	
**Gravidity during last pregnancy**				
1 (primigravida)	102 (89.5)	12 (10.5)	114 (30.0)	0.421
2–4	204 (93.6)	14 (6.4)	218 (57.4)	
5–7	41 (95.3)	3 (4.7)	43 (11.3)	
8–11	5 (100)	0 (0)	5 (1.3)	
**History of Miscarriage (*n*** **= 266)**				
No	198 (94.7)	11 (5.3)	209 (78.6)	0.323
Yes	52 (91.2)	5 (8.8)	57 (21.4)	
**Intention of last pregnancy**				
Unplanned	61 (82.4)	13 (17.6)	74 (19.4)	< 0.001
Planned	291 (95.1)	15 (4.9)	306 (80.6)	
**Term of the last pregnancy during delivery**				
Preterm	26 (83.9)	5 (16.1)	31 (8.2)	0.051
Term	326 (93.4)	23 (6.6)	349 (91.8)	
**Complications during pregnancy or delivery**				
No	327 (93.7)	22 (6.3)	349 (91.8)	0.008
Yes	25 (80.6)	6 (19.4)	31 (8.2)	
**Place of last delivery**				
Home	18 (94.7)	1 (5.3)	19 (5)	0.719
Health facility	334 (92.5)	27 (7.5)	361 (95)	
**Mode of delivery of last pregnancy**				
Cesarean section	57 (93.4)	4 (6.6)	61 (16.1)	0.788
Normal vaginal delivery	295 (92.5)	24 (7.5)	319 (83.9)	
**Infant illness**				
No	316 (94.6)	18 (5.4)	334 (87.9)	< 0.001
Yes	36 (78.3)	10 (21.7)	46 (12.1)	
**Maternal illness after delivery**				
No	334 (94.4)	20 (5.6)	354 (93.2)	< 0.001
Yes	18 (69.2)	8 (30.8)	26 (6.8)	
**Mode of infants’ breastfeeding**				
Not breastfeeding	8 (88.9)	1 (11.1)	9 (2.4)	0.747
Exclusive breastfeeding	339 (92.6)	27 (7.4)	366 (96.3)	
On supplementary feeding	5 (100)	0 (0)	5 (1.3)	

### Bivariate and multivariate regression analysis

On bivariate logistic regression analysis nine socio-demographic and maternity related variables of the mothers were found to predict PPD. However, multivariate analysis revealed only six of them to be independent predictors of postpartum depression. The employment status of the mothers was categorized into employed, unemployed and housewives. Unemployed mothers are those who previously been employed or are potentially employable but currently have no job. Whereas, housewives represent mothers who are married, have no job and are not seeking for employment. The housewives were 0.24 times less likely to develop PPD, as compared to the employed mothers (AOR = 0.24, 95% CI: 0.06–0.97; *p* = 0.046), those mothers who perceived their economic status as low were 13 times more likely to develop PPD as compared to the mothers who had good economic status (AOR = 13.33, 95% CI: 2.66–66.78; *p* = 0.002). Mothers who did not have support from their partner or the father of the baby had higher odds (AOR = 5.8, 95% CI: 1.33–25.29; *p* = 0.019) of experiencing PPD. Participants whose last pregnancy was unplanned were 3.39 times more likely to have postpartum depression (AOR = 3.39, 95% CI: 1.24–9.28; *p* = 0.017). Mothers who experienced illness after delivery were more likely to develop PPD as compared to their counterparts (AOR = 7.42, 95% CI: 1.44–34.2; *p* = 0.016). Finally, mothers from the Southwest of Asmara were found to have higher odds (AOR = 6.35, 95% CI: 1.73–23.23; *p* = 0.05) of being affected by postpartum depression (Table 3).

**Table 3:** Factors associated with postpartum depression

Variable	Category	COR [95% CI]	AOR [95% CI]
**Marital status**	Married	1	1
	Single	3.03 [1.14–8.08]*	0.18 [0.029–1.25]
**Employment status**	Employed	1	1
	Unemployed	4.54 [1.23–16.69]*	0.86 [0.12–5.74]
	Housewife	0.71 [0.22–2.22]	0.24 [0.06–0.97]*
**Perceived economic status**	Bad	12 [3.62–39.7]***	13.33 [2.66–66.78]**
	Average	1.04 [0.36–2.99]	0.88 [0.26–2.94]
	Good	1	1
**Partner support**	Yes	1	1
	No	4.29 [1.57–11.74]**	5.8 [1.33–25.29]*
**Pregnancy**	Planned	1	1
	Unplanned	4.13 [1.87–9.13]***	3.39 [1.24–9.28]*
**Complications during pregnancy or childbirth**	No	1	1
	Yes	3.56 [1.32–9.6]*	2.38 [0.66–8.56]
**Infant illness**	No	1	1
	Yes	4.87 [2.09–11.39]***	1.09 [0.26–4.48]
**Maternal illness after delivery**	No	1	1
	Yes	7.42 [2.87–19.13]***	7.03 [1.44–34.2]*
**Residence**	Northeast Asmara	1	1
	Berikh Sub-zone	1.33 [0.45–3.92]	1.05 [0.26–4.2]
	Southwest Asmara	3.45 [1.3–9.15]*	6.35 [1.73–23.23]**
	Serejeqa Sub-zone	1.31 [0.26–6.65]	1.04 [0.17–6.35]

*p < 0.05, **p < 0.01, ***p < 0.001

## Discussion

This health-facility-based study aimed to asses the prevalence of PPD and factors associated with it among sample of mothers from the Central Region of Eritrea. The prevalence of postpartum depression, using the definitive DSM-5 diagnostic criteria, was found to be 7.4%. This finding was similar to study findings from Sudan [30] and Uganda [31] where the prevalence of PPD was 9.2 and 6.1%, respectively. Higher prevalence rates were also reported from studies in Pakistan (28.8%) [32], Lebanon (21%) [3], Ethiopia (33.3%) [20] and the United States of America (11.4%) [33]. The difference in the reported prevalence of PPD could be due to the difference in the criteria used to diagnose the condition. This study used DSM-5 criteria whereas the other studies used the Edinburgh Postpartum Depression Scale (EPDS) [34] at different cut-off scores. Furthermore, variations in the participants’ ability to identify and report symptoms might have contributed in the observed difference. This problem has been consistently reported when studies use self-administered tools to assess PPD, particularly the EPDS.

### Factors associated with PPD

This study indentified several socio-demographic and maternity-related factors as being associated with PPD. Perceived economic status was a significant predictor of PPD; mothers who perceived their economic status as low were 13.3 times more likely to develop PPD, as compared to those who perceived their economic status as good. This finding is supported by studies conducted in different settings, like Poland [18], Singapore [35], Kenya [36] and Northern California [37]. The impact of poverty on the household in general and women in particular is a well known phenomenon [1, 38]. In the current study setting, childbirth is a well revered and celebrated event. In such cases mothers who have few economic resources may find it stressful to prepare the necessary funds to celebrate the event, which may lead them to PPD.

Studies have consistently reported employment status as a significant predictor of PPD; unemployed mothers being at higher risk than the employed [21, 39, 40]. Similarly in this study employment status of the mother was significantly associated with PPD. On bivariate analysis unemployed mothers were 4.5 times more likely to have depression, compared to the employed mothers. However, on multivariate analysis the housewives had a 74% lower risk of experiencing PPD, compared to the employed mothers. A study conducted in Iran reported contrary findings; housewives were at higher risk of PPD than were employed mothers [41]. The concern of employed mothers about securing their job, on returning to work after delivery, which is not a concern for housewives [42–44], can be a reason for the lower risk of PPD among this group. A study on pregnant Egyptian women found similar findings, with employed women reporting higher levels of depression and anxiety, compared to housewives [22].

Mothers who had no husband/partner support after delivery were 5.8 times more likely to develop postpartum depression than those who had partner support. The effect of partner support or nature of the couple’s relationship has been reported as significant predictors of PPD [36, 42, 45, 46]. Partner support can be viewed from different perspectives. First, partner support can be economic, l such as contributing or covering expenses towards child rearing. Another aspect of the support can be the father’s accepting the baby as his child, particularly in the case of single mothers. This indicates that the above-mentioned factors are most likely to affect single mothers. Finally, mothers may need their partners to share chores, as the additional task of infant care can overwhelm them [47]. In any case, the role of the male partner’s support in preventing and alleviating postpartum depression is commendable [41, 48].

Unplanned pregnancy was another significant factor associated with PPD. In this study mothers whose last pregnancy was unplanned were found to be at higher odds (three times) of developing PPD. Similarly, Barton et al. [49], Sunder et al. [50] and Timothy et al. [33] reported the association between unplanned pregnancy and subsequent PPD in women. However, a study from Hanoi, Vietnam, found no association between unplanned pregnancy and PPD [51]. In this study 42.9% of unintended pregnancies were reported by single mothers as compared to 17.1% of married mothers. This makes interpreting the pathway by which pregnancy intention affects PPD somehow complex, as it could be related to social stigma, inability to bear the responsibility, financial difficulty, or affirming fatherhood.

In this study mothers who became ill after delivery were seven times more likely to experience postpartum depression. Mother’s illness was assed by asking the mother if medication has been prescribed by a physician for the reported problem, to avoid confusion with psychosomatic symptoms. Studies from Uganda [31], and Pakistan [52] have affirmed the link between maternal illness immediately following delivery and postpartum depression. This may be related to the known effect of physical health problems on mental wellbeing [53, 54].

Finally, residence of the mothers was found to be a significant predictor of PPD in this setting. Mothers from Southwest-Asmara were 6.3 times more likely to have PPD as compared to mothers from Northeast- Asmara. The former is an area where relatively affluent people reside, while the latter is a more traditional and slummy residential area. Logically we expected the effect to go the in the opposite direction, with mothers from the slummy area to be more affected by PPD. One possible explanation for this paradox is the culturally determined social support a mother receives during and after delivery in this traditional residence area. Normally the expectant mother is comforted by her neighbors, family members and significant others during and after delivery. This social support ranges from accompanying the pregnant mother to health facility for delivery to washing her and her infant’s clothes. Such social support networks are common in more traditional residential areas where people live a communal life. Viewing the two settings in this way, mothers from Southwest-Asmara would be less likely to receive this kind of social support, than would mothers from Northeast- Asmara. The role of a strong social network in reducing the risk of postpartum depression has been reported in many studies [55–58]. In addition, study findings further indicated that social and cultural norms influence the link between postpartum mental problems and social relationships, by defining what is preferred, expected and exchanged in interpersonal interactions [33]. However, further study is necessary to understand the effects of residence and social interaction in PPD in the current study setting.

### Limitations

This study included only mothers who delivered live-births and who have a healthy child. Mothers with chronic disease, stillbirth, whose infant died after delivery, and mothers with infants in neonatal intensive care units were not included. This limits the ability of this research to healthy mothers with healthy babies. Furthermore, the fact that this was a facility based study the results may not enable us to generalize about the mothers in the general population of the Central Region in which the study was conducted.

## Conclusion

This study found postpartum depression to be a common health problem affecting mothers in the study, which makes it an alarming issue. Occupation, economic status, partner support, unintended pregnancy, maternal physical illness after delivery, and address, were the predictors of postpartum depression among healthy mothers. In this current study setting the factors that influence the occurrence of postpartum depression can be grouped in to two domains; the women’s potential to bear the responsibility of motherhood, and the social support they receive after delivery. Factors such as maternal age, educational level, residence, history of psychiatric illnesses, complications related to childbirth, mode of delivery, place of delivery, and breastfeeding were not associated with postpartum depression. The findings of this study imply the need to introduce an active screening program for PPD in the health facilities as part of the postpartum care. Educating the mothers about the symptoms of PPD will have a significant effect in promoting self-referral for professional help. To increase the effectiveness of screening programs and to reduce pressure in the health services, the primary focus should be targeted towards women at risk of developing PPD.

## Data Availability

The datasets used and/or analysed during the current study are available from the corresponding author on reasonable request.

## Abbreviations

PPD: Postpartum Depression
DSM-5: Diagnostic and Statistical Manual for mental illnesses 5th edition
MDD: Major Depressive Disorders
AOR: Adjusted Odds Ratio
COR: Crude Odds Ratio
CI: Confidence Interval
WHO: World Health Organization
KAP: Knowledge Attitude and Practice
